# A case report of inhibition and severe desnutrition: negative symptoms in resistant schizophrenia

**DOI:** 10.1192/j.eurpsy.2022.2034

**Published:** 2022-09-01

**Authors:** P. Coucheiro Limeres, B. Díez Valle, A. Cerame, C. Lozano Serrano, E. Navas Collado

**Affiliations:** 1 Hospital Universitario José Germain, Psychiatry Department, Leganés, Spain; 2 Hospital Severo Ochoa, Psychiatry, Leganés, Spain

**Keywords:** desnutrition, negative symptoms, resistant schizophrenia

## Abstract

**Introduction:**

The appearance of inhibitory symptoms encompassed in what are known as negative symptoms is part of the usual symptoms of schizophrenia. Sometimes this inhibition reaches a significant severity, so it is essential to know its approach.

**Objectives:**

Case report and literature review regarding the treatment of resistant schizophrenia with a predominance of negative symptoms

**Methods:**

We present the clinical case of a 28-year-old man diagnosed with schizophrenia at 23 years old, whose onset was characterized by delusional ideas of harm (poisoning) and delusions with a mystic-religious theme that lead him to reduce his intake until requiring a first admission for severe desnutrition. Subsequently, after two more admissions, the patient presents selective reduction in food intake, decrease in daily activity and apathy without positive symptoms.

**Results:**

Throughout the treatment, several lines of antipsychotic treatments have been tried at the maximum tolerated dose (haloperidol, oral paliperidone and depot, aripiprazole and clozapine up to a dose of 600 mg). Clozapine resistance required testing various augmentation strategies (Venlafaxine, Lamotrigine and Electroconvulsive therapy) with low results. Finally, to complement the treatment, the patient was transferred to a mid-stay unit where psychosocial treatment with a multidisciplinary approach was started. This has allowed more continuous follow-up and thus a partial improvement of the clinic.

**Conclusions:**

Numerous studies describe numerous augmentation strategies for clozapine-resistant schizophrenia with negative symptoms. However, the results are still inconclusive, needing more research. Meanwhile, we want to highlight the importance of complementing the treatment with psychosocial approaches.

**Disclosure:**

No significant relationships.

